# Characterization of multicellular breast tumor spheroids using image data-driven biophysical mathematical modeling

**DOI:** 10.1038/s41598-020-68324-4

**Published:** 2020-07-14

**Authors:** Haley J. Bowers, Emily E. Fannin, Alexandra Thomas, Jared A. Weis

**Affiliations:** 1 Department of Biomedical Engineering, Wake Forest School of Medicine, 575 N. Patterson Ave., Suite 530, Winston-Salem, NC 27101 USA; 2 Virginia Tech – Wake Forest University School of Biomedical Engineering and Sciences, Blacksburg, VA USA; 3 Comprehensive Cancer Center of Wake Forest Baptist Medical Center, Winston-Salem, NC USA; 4 Department of Internal Medicine, Section of Hematology and Oncology, Wake Forest Baptist Medical Center, Winston-Salem, NC USA

**Keywords:** Breast cancer, Cancer therapy, Computational models, Computer modelling, Differential equations, Numerical simulations

## Abstract

Multicellular tumor spheroid (MCTS) systems provide an in vitro cell culture model system which mimics many of the complexities of an in vivo solid tumor and tumor microenvironment, and are often used to study cancer cell growth and drug efficacy. Here, we present a coupled experimental-computational framework to estimate phenotypic growth and biophysical tumor microenvironment properties. This novel framework utilizes standard microscopy imaging of MCTS systems to drive a biophysical mathematical model of MCTS growth and mechanical interactions. By extending our previous in vivo mechanically-coupled reaction–diffusion modeling framework we developed a microscopy image processing framework capable of mechanistic characterization of MCTS systems. Using MDA-MB-231 breast cancer MCTS, we estimated biophysical parameters of cellular diffusion, rate of cellular proliferation, and cellular tractions forces. We found significant differences in these model-based biophysical parameters throughout the treatment time course between untreated and treated MCTS systems, whereas traditional size-based morphometric parameters were inconclusive. The proposed experimental-computational framework estimates mechanistic MCTS growth and invasion parameters with significant potential to assist in better and more precise assessment of in vitro drug efficacy through the development of computational analysis methodologies for three-dimensional cell culture systems to improve the development and evaluation of antineoplastic drugs.

## Introduction

Recent advances in precision medicine have improved the understanding of breast cancer and have led to the rapid emergence of new antineoplastic therapeutic agents for this patient group^1,2^. Molecularly targeted therapies have significantly increased therapeutic options with improved outcomes for many breast cancer molecular subtypes^3^. With the rapid emergence of new antineoplastic agents, and numerous drugs currently in development, there is an urgent unmet need for the generation of new technologies to provide informative evaluations of their efficiency throughout the stages of drug discovery and development. Traditionally, early stages of drug evaluation are performed using conventional 2D monolayer in vitro cell culture methods and are typically interpreted using drug dose-response curves based on cell viability and fit to a sigmoidal Hill-type model^4^. These observational monolayer drug evaluations have historically served as an essential model for investigating cancer cell behavior and identifying anti-cancer therapeutic efficiency, however these results cannot always be confirmed in preclinical animal trials or clinical trials^5–8^. Increasingly, monolayer cell cultures methods are insufficient for representation of in vivo solid tumors and their complex microenvironment and drug response behavior^9^. Efforts to evaluate drug efficacy for in vitro systems that more appropriately mimic in vivo conditions are being pursued to improve drug development. One example is through the use of three-dimensional (3D) multicellular tumor spheroids (MCTS) as they more closely resemble in vivo solid tumors compared to the much more simplified 2D culture systems, and bridge the gap between conventional monolayer cell culture methods and animal studies^10,11^. 3D MCTS invasion culture systems, consisting of a MCTS embedded within an extracellular matrix (ECM), provide a further enhanced biological model system which recapitulates several architectural and biological behaviors observed in vivo, including cell–cell and cell-extracellular matrix interactions^12–14^. MCTS systems are a more relevant in vitro model compared to monolayer cell culture models and can provide more insight during drug development and subsequent clinical translation. While in vitro MCTS systems represent a simplified approximation to the true nature of in vivo solid tumors, there is a compelling need to study in vitro level models as an important step along the path to translation. In vivo solid tumors exhibit mechanical invasive processes as cells invade into the surrounding ECM during growth and metastasis. Mechanical interactions between cancer cells and their ECM microenvironment can act to regulate cell behavior, function, and response to therapy, however these interactions are often neglected in drug evaluations^15,16^. While in vivo observations of these mechanical interactions are observed on a much longer time scale with more stromal involvement, it has been shown that MCTS systems represent a valuable model system for drug development. Currently, there is a significant limitation in quantitative analysis tools capable of providing mechanistic insight and interpretation of experimental results.

Analysis methodologies for MCTS systems has considerably lagged. Quantifying the growth and response to treatment of MCTS is typically through the use of conventional morphometric analysis techniques using measurements of MCTS length and area to determine growth, shrinkage, or stasis in the presence of drug treatment^17^. These common in vitro analysis techniques are analogous to standard in vivo measurements of the response to therapy performed in the clinic that utilize the response evaluation criteria in solid tumors (RECIST), which is based on measurements of the longest dimension of the tumor from noninvasive patient imaging data^18,19^. While conventional morphometric analysis is capable of identifying overall geometrical changes in MCTS and in vivo solid tumors in response to therapy, the coarseness of the volumetric measurement limits true down-stream mechanistic interpretations of the underlying biophysics of cancer cellular growth and therapeutic response that drives these changes. With the increasing utilization of 3D cell culture methods, including MCTS systems, there is a clear need for analysis tools with more mechanistic insight to biophysically characterize changes in response to drug treatment and uncover more precise evaluation metrics.

Elsewhere, mathematical models have demonstrated utility in quantifying and characterizing the dynamics of cancer, with in silico simulations having been employed to describe MCTS growth^20–23^. Many of these simulations are still driven by conventional morphometric measurements of MCTS, which fail to take into account key changes such as varying cell density and major phenotypic tumor microenvironment (TME) factors limiting their impact. In previous work, we presented a mechanically-coupled reaction–diffusion model capable of describing breast tumor response to neoadjuvant chemotherapy using noninvasive clinical imaging data^24–27^. This model enabled imaging data-driven characterization of tumor changes in response to therapy through fitting biophysical parameters describing cell diffusion and proliferation rate. Further, by coupling the reaction–diffusion model to the surrounding tissue mechanics, we were able to better predict eventual response to therapy. Growth models have also included major phenotypic TME factors including the mechanical interactions between cancer cells and their microenvironment. A highly studied mechanical interaction is the forces exerted by cancer cells on their surrounding environment, known as cellular traction forces, which has gained its popularity in cancer research as it has shown to be a potential biomarker for metastasis^16,29,30^. Cancer cells apply a contractile traction force on their adjacent fibrous matrix elements and act to align and remodel their surrounding ECM which provides paths for cells to invade surrounding tissue^31,32^. It has been shown that these tensile forces originated from the cancer cells facilitates tumor invasion^31,33^. Characterization of these mechanical interactions in MCTS model systems in combination with cellular growth parameters could serve as additional biomarkers of drug responsiveness, which could provide a more complete biophysical characterization of mechanistic changes in drug response assays.

In this study, we propose to extend our mathematical modeling framework to the in vitro MCTS cell culture scale by continuing development of our image data-driven model characterization methods to estimate cellular phenotypic biophysical characteristics of MCTS growth and dynamics. The objective of this work is to propose a modeling framework to functionalize observational in vitro microscopy imaging data to analyze biophysical properties and enable accurate characterization of growth and mechanical interactions of 3D cancer cell culture systems. Our approach incorporates MCTS generation, microscopy imaging, image processing, and mathematical modeling to characterize the mechanistic changes in cellular density and mechanical interactions observed in these MCTS systems. This framework can estimate biophysical parameters of cellular diffusion, rate of cellular proliferation, and the cellular traction force exerted on the surrounding ECM. The objectives of this study are to establish a combined cell culture, imaging, and image analysis protocol to quantify the cellular and mechanical changes in MCTS systems, establish a mathematical model to characterize these changes using phenotypic biophysical parameters, and to identify mechanistic biophysical differences between untreated and treated MCTS systems.

## Methods

### Cell culture

The triple-negative breast cancer cell line, MDA-MB-231, was used to establish the protocol for spheroid generation and were obtained from ATCC, and were maintained in culture according to ATCC recommendations. The cells were labeled using a fluorescent histone H2B lentiviral vector (H2B-GFP, Addgene Plasmid #11680) to facilitate imaging analysis for identifying and estimating cell number. The modified cells were maintained in the same manner as their parental strains.

### Multicellular tumor spheroid generation

**Figure 1:**
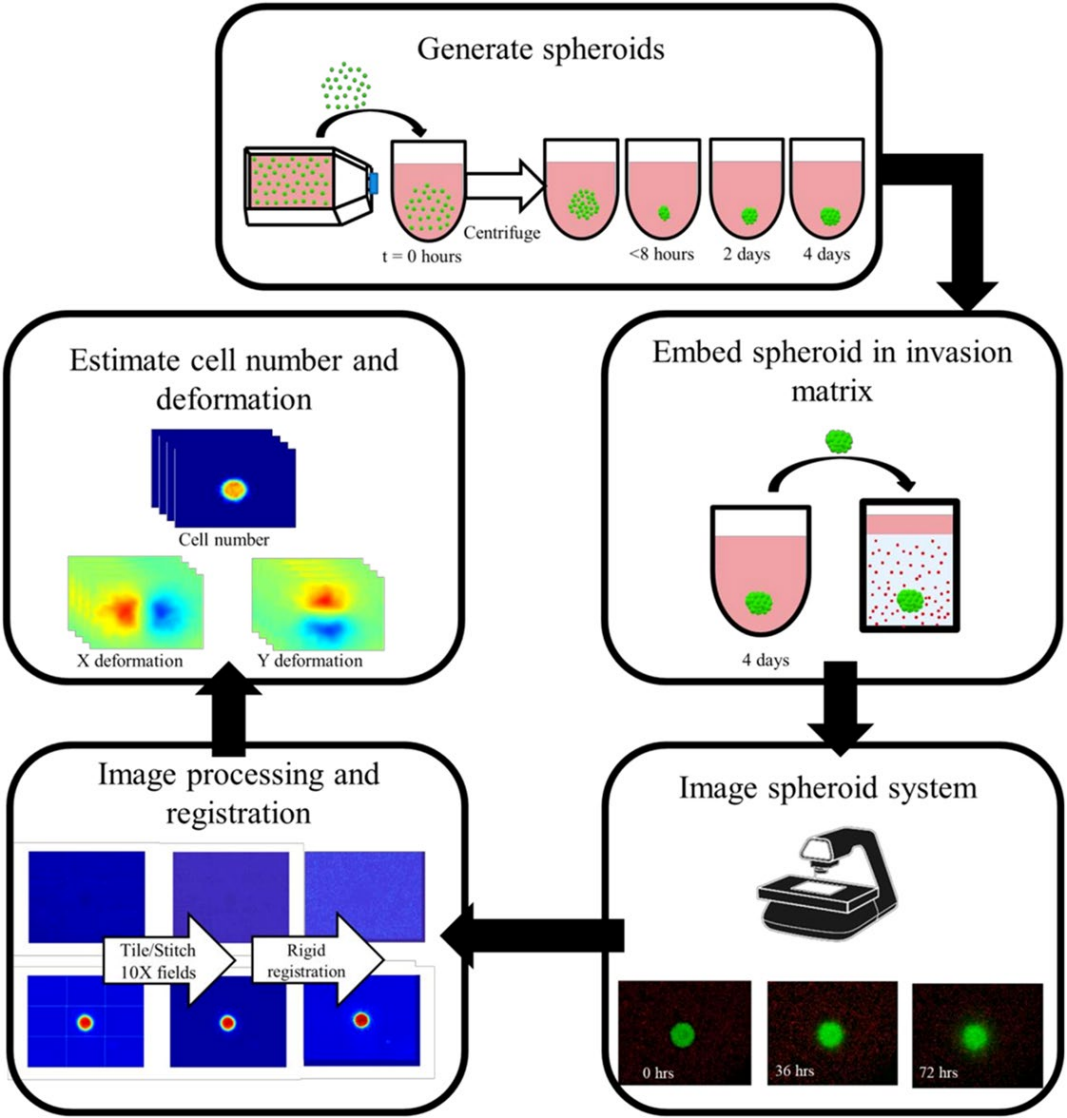
Schematic of MCTS generation and data acquisition protocol. MCTS are generated using ultra-low attachment microplates which are cultivated for 4 days. Once formed, MCTS are embedded into a collagen invasion matrix containing fluorescent microspheres to track extracellular matrix deformation. Time-lapse microscopy imaging of MCTS systems occurred over a 72-h time span. Images were tiled, stitched and rigidly registered before estimating cell number and X and Y deformation for the model.

MCTS were generated using the liquid overlay technique^34^ using CellCarrier spheroid ultra-low attachment 96-well microplates (Perkin Elmer). A total suspension volume of 200 μl containing 5,000 cells, cell-specific medium and 0.35 mg/ml concentration of Matrigel (Corning) was added to wells. To initiate spheroid formation, the microplate was centrifuged in a swinging bucket rotor at 300×g for 5 min. Cells were cultivated for 4 days to allow for spheroid self-assembly. Figure 1 shows a schematic describing the time course of MCTS generation.

### Invasion matrix

A collagen solution made up of neutralized collagen type I (2.25 mg/ml, Corning) and is co-polymerized with 2 μm red fluorescent microsphere beads (FluoSpheres™ Carboxylate-Modified Microspheres, 580/605, 2 × 10^6^ beads/ml) used to track extracellular matrix deformation. The spheroid generation plate was chilled on ice for 15 min to pre-chill wells^22^. 200 μl of collagen solution was placed into a flat bottom 96-well ultra-microplate (Perkin Elmer, ULA CellCarrier-96 Ultra Microplates) with an optically-clear cyclic olefin bottom for optimal imaging acquisition. Once chilled, 25 μl containing the spheroid was removed from the spheroid generation plate and was added to the collagen solution (final collagen concentration of 2 mg/ml) and gently pipette mixed. Once all replicates were complete, the plate was allowed to polymerize at room temperature for 1 h followed by polymerization at 37 C for 1 h. Once completely polymerized, 100 μl of medium was overlaid in each well. A schematic representation of spheroid embedding into the invasion matrix is found in Fig. 1.

### Drug treatment

At 24 h following embedding of MCTS in invasion matrix, spheroids were treated with nanoparticle albumin-bound paclitaxel (Abraxane) at a concentration of 400 nM. MCTS systems were exposed to the drug for 72 h. MCTS systems (treated and untreated) underwent media changes every 72 h.

### MCTS imaging and image processing

While methodological descriptions for imaging and imaging processing steps follow, additional materials further describing and documenting the steps are available as supplementary materials. Briefly, time-lapse fluorescent microscopy imaging of both untreated and treated MCTS systems was used to characterize the changes in growth and dynamics. Imaging began 24 h after embedding MCTS in collagen and continued every 12 h for a total imaging time of 72 h. MCTS were imaged using a fully-automated time-lapse fluorescent microscope (EVOS FL Auto 2 Cell Imaging System, Invitrogen) with an on-stage incubator controlling C0_2_ (5%), O_2_, temperature (37˚C), and humidity (EVOS Onstage Incubator, Invitrogen). Z-stack images of the MCTS and surrounding ECM were acquired with a 3 by 3 imaging grid using a 10X objective for an overall 500 × 500-micron field of view with pixel sizes of approximately 5 µm. Images were acquired in two co-registered fluorescence color channels, with the green channel (GFP) to image the MCTS and the red channel (Texas Red) to image the fluorescent microbeads. Figure 1 shows the workflow of data acquisition, image processing, and registration, the necessary steps to prepare the observational microscopy images to estimate cell number and deformation.

Microscopy images were compiled and stitched using a customized fully-automated tiling, stitching, registration, and segmentation software written in MATLAB (Mathworks, Natick, MA). Z-stacks for each 10X field were compiled and stacked to create a 3D image of the field. Initial position data for each field was extracted from the microscopy acquisition data and fields were stitched using rigid registration based on intensity correlation of the overlap between images. 3D point clouds were then constructed to represent the centroid position of the fluorescent beads using the binary image, and used to rigidly register each serial time point image to the initial time point using an iterative closest point algorithm. Uncommon z-planes between the time points due to physical microplate repositioning between imaging acquisitions were eliminated. To reduce dimensionality, maximum intensity projection images of the registered bead and MCTS images were used for subsequent image processing and analysis. MCTS pixel fluorescent intensity was normalized by identifying the peak intensity for the top 0.01% of pixels among all wells in the plate and across all time points, and normalizing each image to a scale of 0–1. This normalization defines the cell number carrying capacity for the model with the assumption of a linear relationship between fluorescent intensity and cell number. Regions-of-Interest (ROIs) for each MCTS were identified at each time point using a fully-automatic threshold-based segmentation method. Bead images were non-rigidly registered to the previous time point using multi-resolution free-field deformations based on multi-level B-splines to obtain the observed bead deformation field^35,36^.

### Biophysical model of MCTS growth and deformation

The biophysical MCTS growth model is based on previous work from our group^24,27^ developed at the in vivo clinical scale and modified for use at the in vitro MCTS scale. This study incorporates the same fundamental model used in our previous work with minimal modifications allowing for it to be used at the in vitro scale. The governing coupled partial differential equations are shown in Eqs. (1)–(3) below:

1 $$ \frac{{\partial N\left( {\overline{x},t} \right)}}{\partial t} = \nabla \cdot \left( {D\nabla N\left( {\overline{x},t} \right)} \right) + kN\left( {\overline{x},t} \right)\left( {1 - \frac{{N\left( {\overline{x},t} \right)}}{\theta }} \right) $$

2 $$ D = D_{0} e^{{ - \gamma \sigma_{VM} \left( {\overline{x},t} \right)}} $$

3 $$ \nabla \cdot G\nabla \overset{\lower0.5em\hbox{$\smash{\scriptscriptstyle\rightharpoonup}$}} {u} + \nabla \frac{G}{1 - 2v}\left( {\nabla \cdot \overset{\lower0.5em\hbox{$\smash{\scriptscriptstyle\rightharpoonup}$}} {u} } \right) - \lambda \nabla N\left( {\overline{x},t} \right) = 0 $$

Equation (1) models the rate of change of cell number, *N* $(\stackrel{-}{x},t)$, at a given time and location as the sum of random cell diffusion and logistic growth, where *D* (μm^2^/h) is the local cellular diffusion coefficient of tumor cells in the presence of mechanical stress, *k* (h^−1^) is the cell proliferation rate, and *θ* is the cell carrying capacity. Equation (2) couples Eq. (1) to the surrounding tissue stiffness and describes cell diffusion coefficient as a modification of the global cell diffusion coefficient, *D*_*0*_, through distortional (von Mises) stress, *σ*_*VM*_, and an empirically derived coupling constant, *γ*. Equation (3) describes linear elastic, isotropic mechanical equilibrium with an external expansive force determined by changes in cell number and a biophysical traction force coefficient, *λ*, which governs the response of the displacement vector, *u*, to tumor cell growth. *G* represents shear modulus and is defined as *G* = *E*/2(1 + *v*) where *E* represents Young’s modulus and *v* represents Poisson’s ratio. A uniform Young’s modulus of 2 kPa is assigned to the ECM, based on the assumption of uniform invasion matrix density throughout. In our previous work, the mechanical coupling constant, *λ,* was an empirically-derived coupling constant. In this work, model-to-data fitting of *λ* is used to estimate the observed mechanical interactions in the MCTS system. The Young’s modulus of the spheroid core is unknown but assigned as an order of magnitude higher than the surrounding environment with the assumption that the spheroid core is stiffer than the collagen invasion matrix. We return to these assumptions in the Discussion section. Poisson’s ratio is assumed as 0.45 representing the near-incompressible nature of the hydrogel system and avoiding numerical instabilities due to incompressibility within a linear elastic model solution^17^. Time stepping was assigned with Δt = 0.125 h for a duration of 12 h for each time point, for a total of 72 h (hour 96 to hour 24). Finite element meshes were constructed to represent the observed microscopy field of view domain and were composed of three-node triangular elements with an average edge length of 45 μm. The equations are solved using the Galerkin method of weighted residuals on triangular finite elements described by standard linear Lagrange basis functions using a fully explicit forward Euler method. We assume that the MCTS growth takes the form of a reaction diffusion logistic growth model, a commonly studied model for estimating the growth of tumors and avascular spheroids. We utilize a first-order simplifying approximation by neglecting the convective velocity of cellular mitosis under the assumption that the time scale of tumor growth is sufficiently long such that convection motion driven by cell mitosis is minimal relative to diffusive motility^28^.

### Parameter estimation

**Figure 2:**
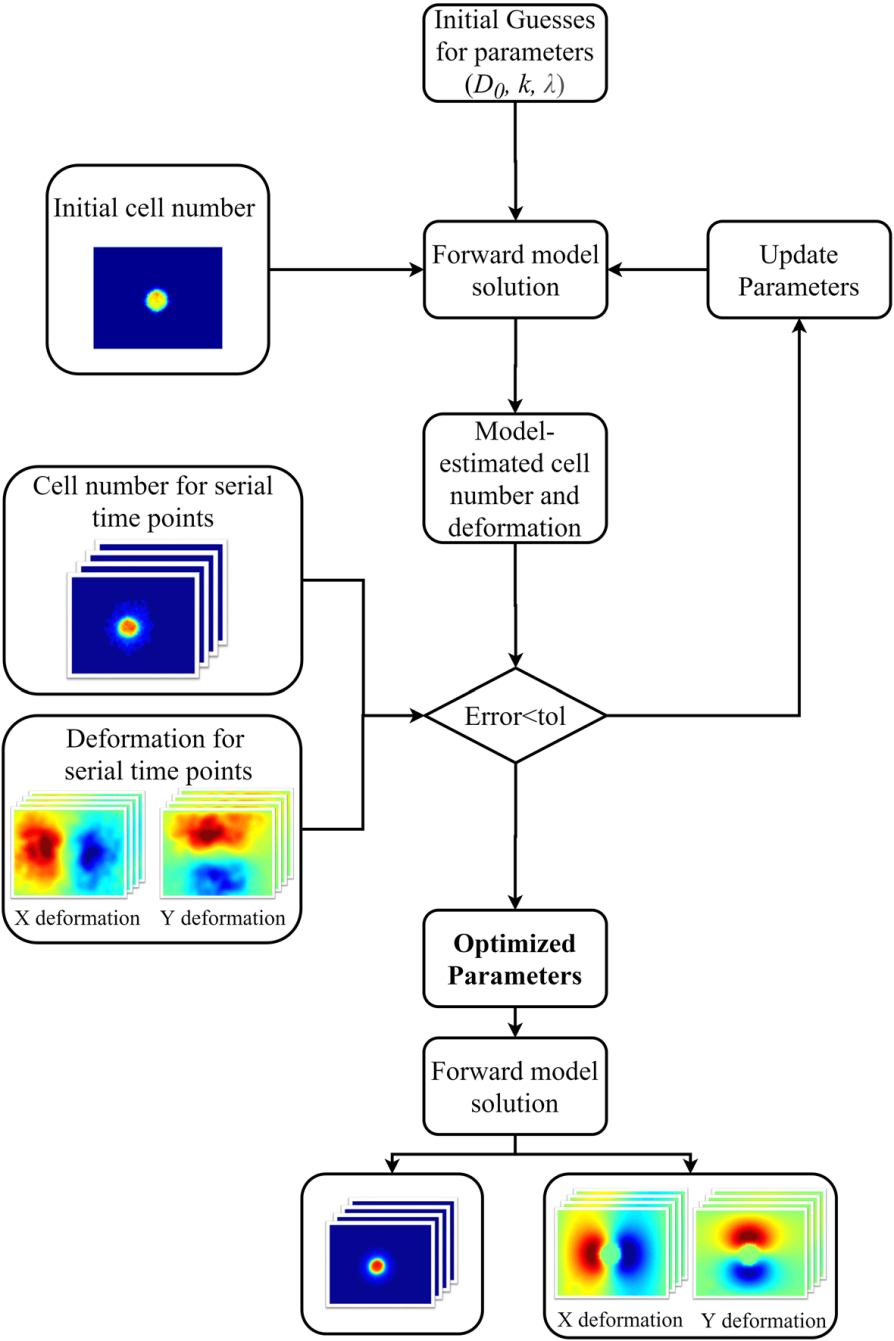
Model schematic of microscopy image-driven biophysical mathematical model parameter estimation. The model is used to build a map of cellular density, X deformation, and Y deformation and parameters are iteratively optimized until the error is minimized below the preset tolerance. The fit parameters are then used to project the model forward in time to obtain the model estimated images.

Figure 2 shows the modeling approach for characterizing MCTS growth and mechanical interactions. A Levenberg–Marquardt least-squares non-linear optimization was used to estimate the proliferation rate (*k*), cellular diffusion coefficient (*D*_0_), and the traction force coefficient (*λ*) between each imaging time point. Model parameters are assumed as piecewise continuous between each imaging time point over the observed time scale as linear uniform time invariant parameterization was determined as inadequate for characterizing MCTS systems. Modeling the changes between the observed time points allowed us to capture the dynamic changes of these biophysical parameters. Model parameters are assumed to be homogenous throughout the domain.

The objective function that was minimized is the sum-squared error between the observed and model-estimated spheroid cellularity, and the observed and model-estimated traction force displacement. Iterative parameter estimation was terminated based on the following convergence criteria: (a) absolute objective function value less than 1 × 10^–4^, (b) relative change in the objective function between iterations less than 1 × 10^–2^, (c) maximum of 500 iterations, or (d) maximum of 1,000 function evaluations. We note that relative objective function change was the typical convergence condition.

To improve inverse parameter optimization performance, we use an iterative sequential method to independently solve for the model parameters. Our sequential method involves solving the inverse problems in steps by fitting the model parameters between two sequential time points. Initially we use a cell number objective function to fit parameters of *D*_0_ and *k*, with an arbitrary assigned λ. We then fit *λ* using a displacement objective function holding the optimized first iteration *D*_0_ and *k* parameters fixed. We then repeat the process until the final model parameters are converged, which occurred in two iterations in all cases. In data not shown, we validated our parameter estimation methodology using synthetic in silico MCTS systems subjected over a dynamic range of parameters and found convergence of the inverse problem for parameter estimation to the correct set of parameters.

Spheroid ROIs and X and Y bead deformation at each time point were interpolated onto the mesh and used as inputs for the model. We derive a first-order approximation for spheroid cell density using a linear relationship between cell density and fluorescent intensity, with a maximal carrying capacity, *ϴ*, of 1 set from normalization of spheroid fluorescent intensity described above.

## Results

**Figure 3:**
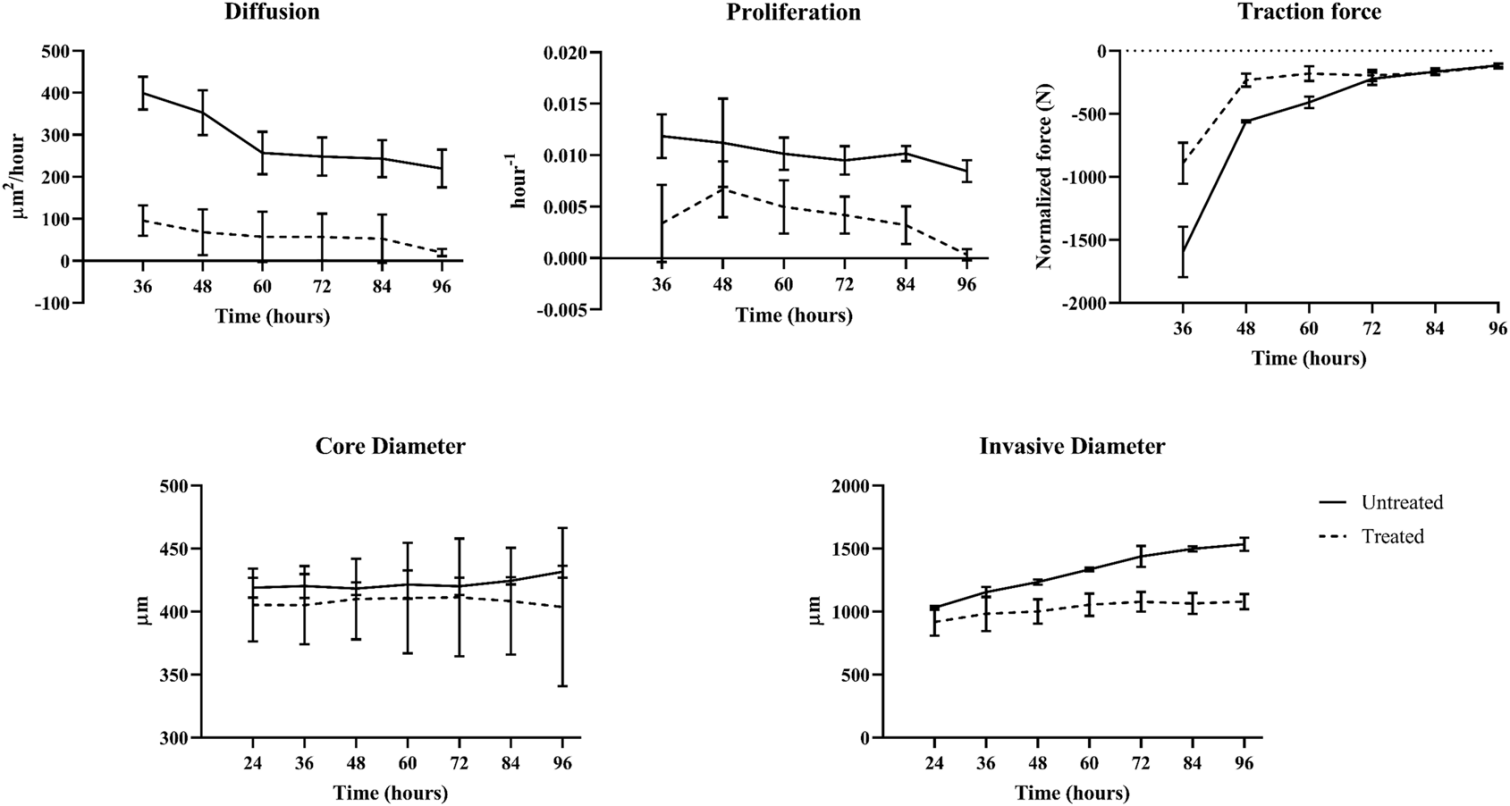
(Top) Preliminary data results with model estimated biophysical parameters of diffusion, proliferation, and traction force and (bottom) conventional morphometric assessment methods. As previously described, parameters *D*_*0*_, *k*, and *λ* were fit for each untreated and treated MCTS system over the time course. The model fit parameters are able to describe the underlying biophysics driving MCTS changes in response to paclitaxel treatment. The conventional morphometric assessment methods of core diameter and invasive diameter can describe overall growth, shrinkage, or stasis of MCTS, but are unable to infer mechanistic biophysical interpretation.

**Figure 4:**
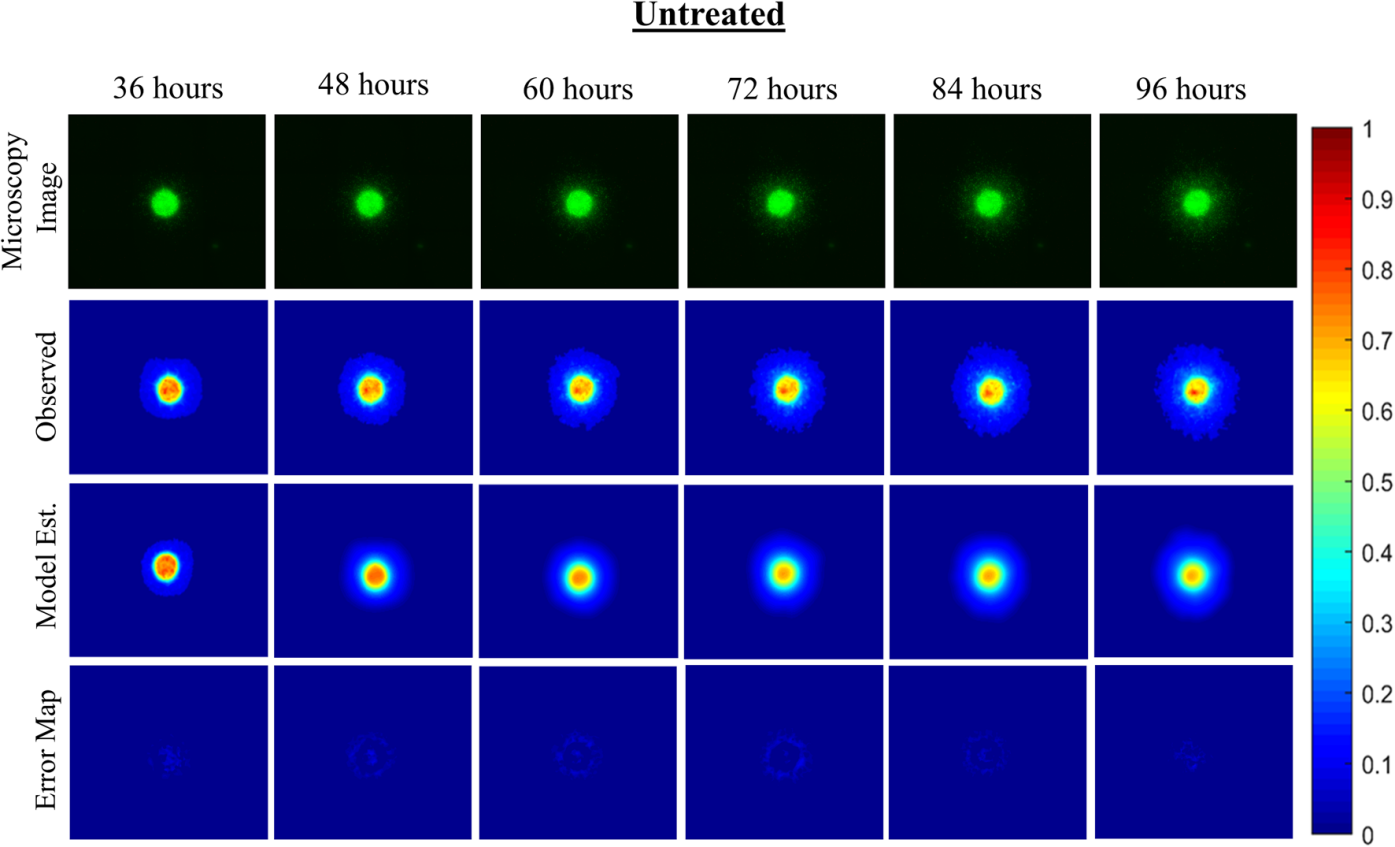
Observational microscopy images, model characterization of cellular density, and error maps between the observed and model-estimated cellular density for one representative untreated MCTS system is shown. Processed observational microscopy images show cellular density on a scale from 0 to 1. Cellular density is estimated using our computational model and is used to estimate parameters of cellular diffusion and proliferation between each imaging time point. Error maps show model-data misfit of cellular density.

**Figure 5:**
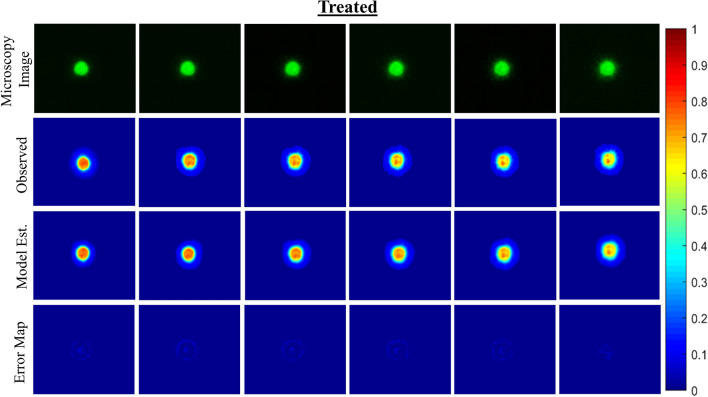
Observational microscopy images, model characterization of cellular density, and error maps between the observed and model estimated cellular density for one representative treated MCTS system is shown. Processed observational microscopy images show cellular density on a scale from 0 to 1. Cellular density is estimated using our computational model and is used to estimate parameters of cellular diffusion and proliferation between each imaging time point. Error maps show model-data misfit of cellular density.

**Figure 6:**
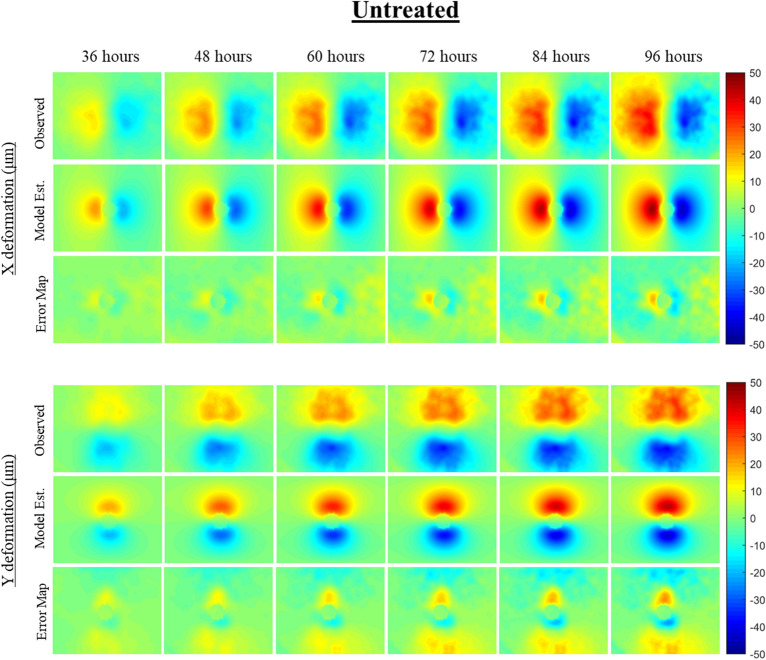
The model characterization of cellular traction force for one representative untreated MCTS system is shown. X and Y deformation field images are determined based on non-rigid registration of observational microscopy imaging of fluorescent microbeads over time. Using the model-fit cellular density parameters and observational deformation field images in combination with our computational model, a cellular traction force parameter is estimated between each imaging time point. The figure reflects the cumulative cellular traction force overtime in both and X and Y displacement directions. Error maps show the model-data misfit for both X and Y deformation.

**Figure 7:**
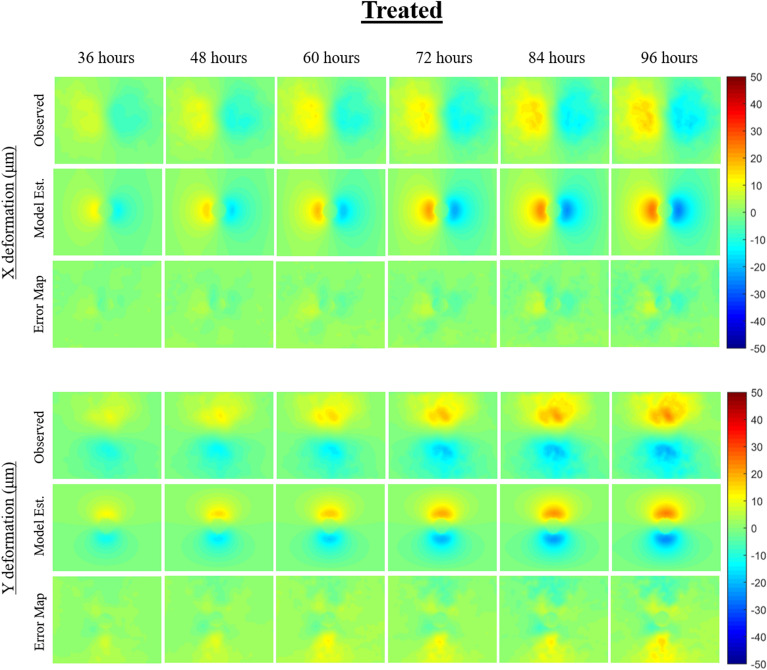
The model characterization of cellular traction force for one representative treated MCTS system is shown. X and Y deformation field images are determined based on non-rigid registration of observational microscopy imaging of fluorescent microbeads over time. Using the model-fit cellular density parameters and observational deformation field images in combination with our computational model, a cellular traction force parameter is estimated between each imaging time point. The figure reflects the cumulative cellular traction force overtime in both and X and Y displacement directions. Error maps show the model-data misfit for both X and Y deformation.

Six MCTS systems (*n* = 3 untreated, *n* = 3 treated) were imaged and analyzed using our mathematical model. Figure 3 displays our data results for our model estimated biophysical parameters of cellular diffusion, cellular proliferation rate, and cellular traction force over the observed time course. Our mechanically-coupled reaction diffusion model was capable of describing MCTS growth and traction force for both treated and untreated conditions. Figure 3 compares our model-estimated biophysical parameters to conventional morphometric techniques. Over time the MCTS core density is observed to reduce which we hypothesize is due to a combination of the diffusive motility (in which cells dissociate at a higher rate than proliferation) along with the avascular nature of the MCTS core. Imaging data and model predictions for MCTS systems from an untreated and treated representative MCTS systems are shown in Figs. 4, 5, 6 and 7. The mechanically-coupled reaction–diffusion model is used to estimate parameters of diffusion, proliferation rate, and mechanical traction forces by fitting the model to pairs of observational MCTS microscopy images. The model-based parameters are used to characterize untreated and treated MCTS systems to distinguish between the two groups. Figures 4 and 5 display and compare cellular density observation and model-estimated data over the imaging time course for representative untreated and treated MCTS systems. Qualitatively, the untreated MCTS systems show cellular invasion into the surrounding microenvironment whereas as compared to hindered cellular invasion in the treated MCTS systems. Compared to observed cellularity data, our model underestimates MCTS core cellularity once cellular invasion into the ECM begins. Figures 6 and 7 display and compare X and Y deformation observation and model-estimated data over the imaging time course from representative untreated and treated MCTS systems. As expected, we observe more deformation in untreated MCTS systems compared to treated MCTS systems. In both untreated and treated MCTS systems, peak deformation is observed adjacent to the MCTS edge with a reduction in deformation in the radial direction. We also observe the bulk of deformation changes during the earlier time points over the imaging time course with the differences in deformation minimizing in later time points. The bead deformation field image is obtained through non-rigid registration of fluorescent bead images, and the model is used to estimate *λ*, to characterize the underlying biophysics of cell-ECM mechanical interactions. Qualitatively, the treated MCTS deformation field map exhibits a significant decrease in bead displacement relative to untreated MCTS. Statistical analysis was performed to determine the ability of the model to characterize treatment effects on MCTS systems using biophysical parameters. An unpaired parametric *t*-test was used for each parameter at each time point with a significance level, alpha of 0.05. The mean, standard deviation and statistical significance for each parameter are reported in Table 1. As shown in Table 1, the model was able to characterize treatment-induced differences in MCTS growth and mechanical interaction biophysical parameters over the time course.

**Table 1:** Mean and standard deviations for each model parameter and time point.

Time point (hours)	Diffusion		Proliferation		Traction force	
	Untreated	Treated	Untreated	Treated	Untreated	Treated
36	399.46 ± 31.99	95.79 ± 29.36***	1.18E−2 ± 1.73E −3	3.37E−3 ± 3.07E −3*	− 1596.31 ± 163.03	− 892.13 ± 133.93**
48	352.7 ± 43.77	68.04 ± 44.57***	1.12E−2 ± 3.52E−3	6.69E−3 ± 2.21E−3	− 560.88 ± 6.28	− 232.76 ± 42.75***
60	256.72 ± 41.08	57.33 ± 48.54*	1.01E−2 ± 1.28E−3	4.96E−3 ± 2.12E−3*	− 408.97 ± 36.36	− 181.65 ± 48.96**
72	248.27 ± 37.28	56.67 ± 45.43**	9.5E−3 ± 1.14E−3	4.18E−3 ± 1.47E−3*	− 222.96 ± 40.28	− 195.46 ± 35.75
84	243.17 ± 36.16	52.84 ± 47.09*	1.02E−3 ± 5.95E−4	3.20E−3 ± 1.50E−3**	− 166.50 ± 21.83	− 170.20 ± 14.36
96	219.83 ± 36.91	19.87 ± 7.15**	8.45E−3 ± 8.64E−4	3.20E−4 ± 4.53E−4***	− 115.15 ± 11.28	− 121.20 ± 15.06

*Denotes statistical significance with *p* < 0.05, **p < 0.01, ***p < 0.001.

Our results show promise in describing MCTS systems using *D*_*0*_, *k*, and *λ*. To compare our biophysical model-based biomarker analysis to conventional MCTS analysis we assessed conventional morphometric analysis of core and invasive diameter. We defined the core diameter using the maximum intensity projection image at the initial time point with a threshold selected for fluorescent intensity to define the core diameter. The longest dimension of the total MCTS ROI was defined as the invasive diameter. Figure 3 shows model estimated biophysical parameters and the conventional morphometric analysis over the observed time course. We found that the core diameter was not able to differentiate between treated and untreated conditions with no significant differences throughout the treatment time course. The traditional morphologic measurement of invasive diameter was able to distinguish between the treated and untreated MCTS systems, but is only statistically significant following 48 h of growth and invasion. Comparatively, the combination of our model fit parameters *D*_*0*_, *k*, and *λ* better characterize the biophysical changes between untreated and treated MCTS systems with increased sensitivity throughout the entire time course.

## Discussion

In this work, we modified our previously published mechanically-coupled reaction–diffusion model by extension from the in vivo clinical scale to describe in vitro MCTS growth, invasion, and mechanical interaction within extracellular matrix. We show that biophysical model parameters of cell diffusion, proliferation, and cellular traction force can be reliably estimated from observational microscopy imaging of MCTS growth and mechanical interactions. These model-based quantitative biomarkers indicate the underlying biophysics driving growth and invasion into the surrounding ECM and reveal important biological insights through the use of observational imaging data alone. Importantly, the model-based framework was able to distinguish between treated and untreated MCTS systems, validating that our modeling framework is able to characterize both growth and treatment-related parameters. We found significant differences in model parameters of diffusion, proliferation, and cellular traction force between untreated and treated MCTS spheroids. In this study, we use the antineoplastic therapeutic nab-paclitaxel, a taxane-based chemotherapeutic antimicrotubule agent. Taxanes stabilize microtubules against depolymerization, blocking the cell cycle in G1 or M phases and preventing cell division and triggering cell cycle arrest and mitotic catastrophe^37^. This results in reduced proliferation, which is reflected in our modeling framework through significantly lower proliferation rates observed for treated MCTS systems. Further, microtubule cytoskeletal networks are crucial for transducing and regulating mechanical signals, with cellular traction force generated through microtubule depolymerization^38^. This mechanism is reflected in our modeling framework through significant attenuation in the traction force parameter at early time points. At the later time points, there is a significant reduction in the amount of cellular traction force which may be an effect of the ECM stiffening. In untreated MCTS systems, we see a substantial amount of initial traction force, but the traction force is significantly lower in MCTS systems treated with nab-paclitaxel. The mechanistic validation of our modeling framework through the use of a widely-studied antineoplastic agent with well-characterized mechanisms of action provides significant promise for the use of our model to characterize biophysical mechanistic treatment effects.

Here, we show characterization of the dynamic changes in mechanistic biophysical properties that enhances the available information from traditional drug sensitivity assays without requiring significant changes to current observational imaging protocols. In the current state of conventional drug sensitivity screening, morphometric measurements and cell counts reflect the eventual downstream consequences of treatment (cytostatic or cytotoxic effects) but obfuscates the underlying mechanisms that are driving observed tumor growth and therapeutic responses. While we show that conventional assessment methods (e.g. spheroid core and invasive diameter) may eventually yield statistically significant changes in response to treatment that reflects observable cytotoxic effects, biophysical characterization methods as developed in this study provide advantages in providing mechanistic insight. The spheroid core diameter was unable to distinguish between the treated and untreated MCTS systems throughout the entire time course. The core diameter also showed a slight reduction in MCTS cell density over the time course which we hypothesize could be due to diffusive motility and the avascular nature of MCTS systems. Due to the mechanical invasion motility process, cells dissociate from the MCTS core at a faster rate than they are proliferating leading to a slight core reduction. Additionally, as the MCTS systems are an avascular system, the internal cells of the MCTS core may receive restricted oxygen and nutrient penetration potentially leading to cellular senesce and/or apoptosis. The spheroid invasive diameter was able to distinguish between the treated and untreated MCTS systems at later time points, however mechanistic interpretation is limited with failure to describe the underlying biophysics which drive these observed morphometric changes.

While the results of this study are promising, there are several limitations. In our model we assumed homogeneous mechanical stiffness throughout the extracellular matrix domain with the heterogeneity of collagen matrix stiffness not considered in this work. Cancer cells have been shown to induce ECM remodeling through deposition, crosslinking, and alignment of ECM collagen fibers which is known to stiffen the ECM, however accurate methods to quantify the bulk heterogeneity of mechanical stiffness in ECM in vitro systems are challenging. It would be of interest to develop methods to quantify stiffness throughout MCTS systems over time. By incorporating more accurate mechanical stiffness assumptions, we could improve our spatiotemporal model fits. Incorporating this heterogeneity would increase the complexity of the proposed model, requiring additional experimental data to characterize the stiffness. Additionally, in this work, we only characterize the MCTS systems using 2D analysis. While acquired images are 3D, in this first-order analysis we dimensionally-reduced our input data to 2D in order to simplify the initial validation of our modeling framework to describe biophysical changes in response to treatment. With further model development, in future studies we will characterize these biophysical parameters in 3D. Despite these limitations, our model allowed for the mechanistic characterization of biophysical changes in MCTS systems, improving upon current conventional morphometric assessment methods.

While in this work we restricted our analysis to biophysical parameters of proliferation, diffusion, and traction forces, this mathematical analysis framework is generalizable and has the ability to be extended to evaluate additional biophysical phenomena. Our model has the potential to additionally account for other phenotypic biophysical TME factors affecting growth and therapeutic response, including the addition of active migration/motility through haptotaxis along stiffness gradients, cellular metabolism regulated by spatial oxygen gradients, and collagen fiber remodeling and alignment. Our methodology also shows promise in the ability to describe cell population-specific and drug-specific biophysics by evaluating various breast cancer cell lines and therapeutic agents. Our modeling framework may also be applied to assessment of 3D breast cancer organoid models using patient-derived cells. Characterizing mechanistic biophysical parameters could enable patient-specific therapeutic selection by identifying patient-specific biophysical phenomena driving tumor growth or reflecting particular therapeutic vulnerabilities.

This integration of microscopy imaging, biophysics, and mathematical modeling has the potential to impact many areas of research pertaining to dynamics of cancer growth and therapeutic response including cell line observations, drug sensitivity assays, anti-cancer drug development, and patient-specific drug selection and prediction of response. Ultimately, pre-existing cellular biophysics dictates individual phenotypic observations of cancer growth and response to therapy; yet conventional microscopy imaging assessment methods are relegated to monitoring the conclusions of these phenotypic responses and not their underlying causes. The coarseness of these outcome measures complicates efforts to understand the mechanistic effects of drugs or the specific driving biological factors in patient-derived samples as many different combinations and levels of various biophysical phenomena may yield similar final observed responses. In this work, we demonstrate the utility of a microscopy imaging-data driven modeling approach to quantify cellular biophysics based on fitting longitudinal observations to biophysical models in an important step towards precision evaluation.

## Supplementary information

Supplementary information
