# Supplementary material for: Characterization of multicellular breast tumor spheroids using image data-driven biophysical mathematical modeling
Source: Sci Rep. 2020 Jul 14;10:11583. doi: 10.1038/s41598-020-68324-4 (PMC7360601; doi:10.1038/s41598-020-68324-4)
Supplement: Supplementary file 1 — Supplementary information [file 41598_2020_68324_MOESM1_ESM.docx]

Characterization of multicellular breast tumor spheroids using image data-driven biophysical mathematical modeling

Haley J. Bowers^1,2^, Emily E. Fannin^1^, Alexandra Thomas^3,4^, Jared A. Weis*^1,2,3^

^1^Department of Biomedical Engineering, Wake Forest School of Medicine, ^2^Virginia Tech – Wake Forest University School of Biomedical Engineering and Sciences, ^3^Comprehensive Cancer Center of Wake Forest Baptist Medical Center, ^4^Department of Internal Medicine, Section of Hematology and Oncology, Wake Forest Baptist Medical Center

^*^Corresponding Author:

Jared A. Weis, PhD

575 N. Patterson Ave.

Suite 530

Winston-Salem NC, 27101

Email: jweis@wakehealth.edu

## Supplemental Material

**S.1. Bead deformation fields**

Observed bead field images were used to estimate ECM deformation due to traction forces. The observed bead deformation field is estimated by non-rigidly registering the acquired bead image at a time point to the previous time point using multi-resolution free-field deformations based on multi-level B-splines. Supplemental Figure 1 shows acquired bead field images before and after non-rigid registration. Our non-rigid registration enforces smoothing, capturing a local area-based average of induced deformation. The registration captures general deformation trends well, however as expected, we observe some minor local variability due to smoothing. In particular, there tends to be a higher degree of bead registration misfit closer to the MCTS edge, where the bead deformation is highest.


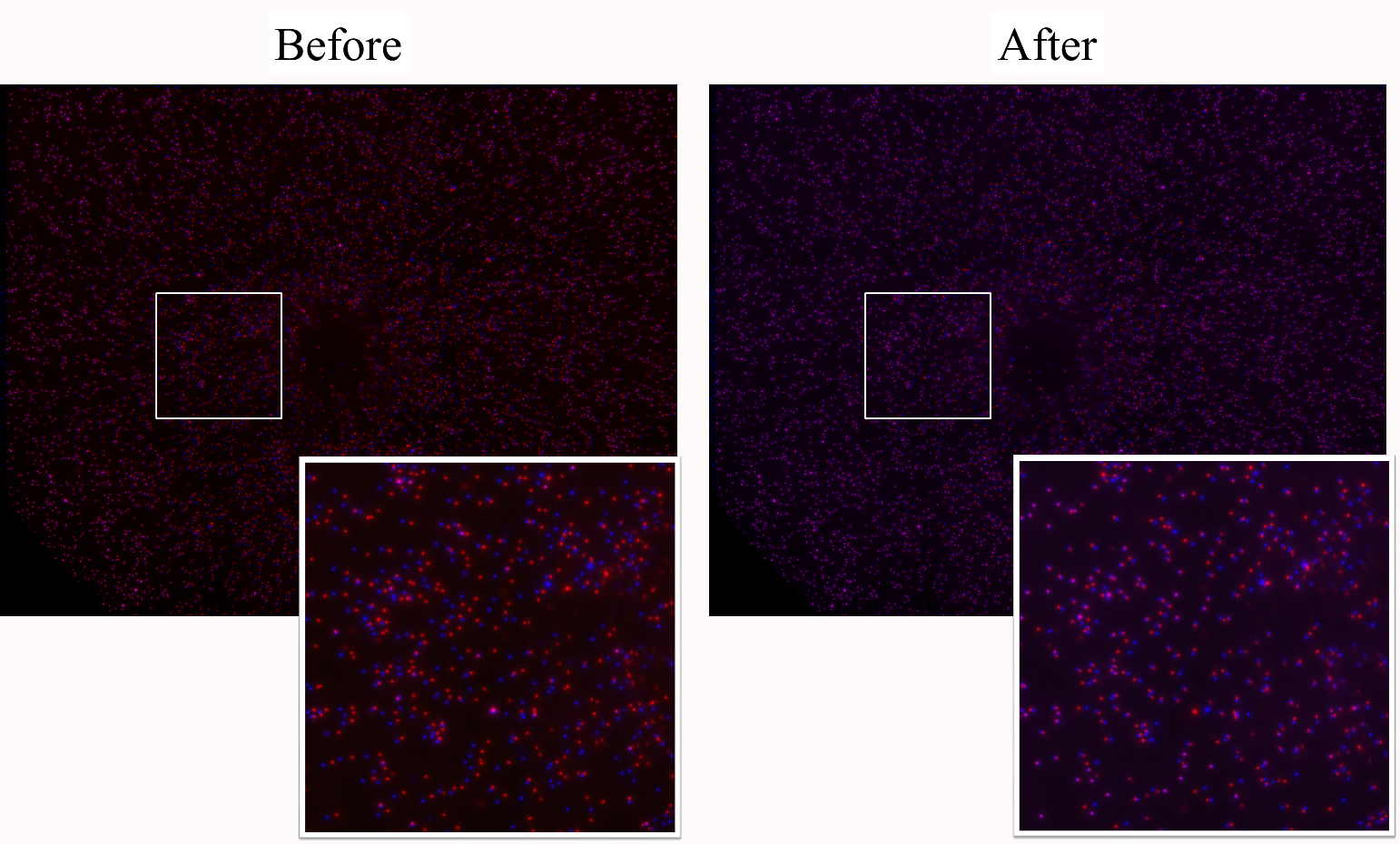


**Supplemental Figure 1:** Images of bead fields before and after non-rigid registration of bead fields to capture the observed deformation. The 24-hour time point bead field is shown in blue and the 36-hour time point bead field is shown in red. In the enlarged ‘before’ image (left) we observe inward displacement towards the MCTS between 24 and 36 hours due to cellular traction forces. In the ‘after’ non-rigid registration image (right), magenta colored beads represent the registration (overlay of registered red beads and blue beads). In general, the non-rigid registration bead deformation estimation procedure accurately captures the deformation field with some minor local registration misfit due to applied smoothing.

**S.2. MCTS imaging and image processing: Time-lapse image acquisition**

All fluorescent microscopy images were acquired with a 3 by 3 imaging grid using a 10X objective. Within this grid, 50 z-stacks were acquired in two co-registered fluorescence color channels, with the green channel (GFP) to image the MCTS and the red channel (Texas Red) to image the fluorescent microbeads. Once images were acquired, they were compiled and stitched using a customized fully automated process. Recorded imaging position data for each of the 9 fields was extracted from the acquisition data and were used to initially configure the 3 by 3 grid. Images were acquired with an overlap between the fields, and the overlap was used to stitch the images by using rigid registration of the individual image fields based on the intensity correlation coefficient. Supplemental Figure 2 shows an example of the 3 by 3 imaging grid with the overlap highlighted prior to stitching along with the final stitched image.


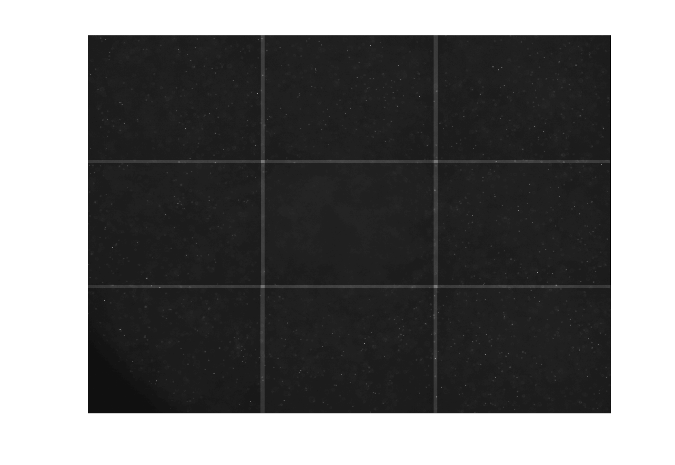

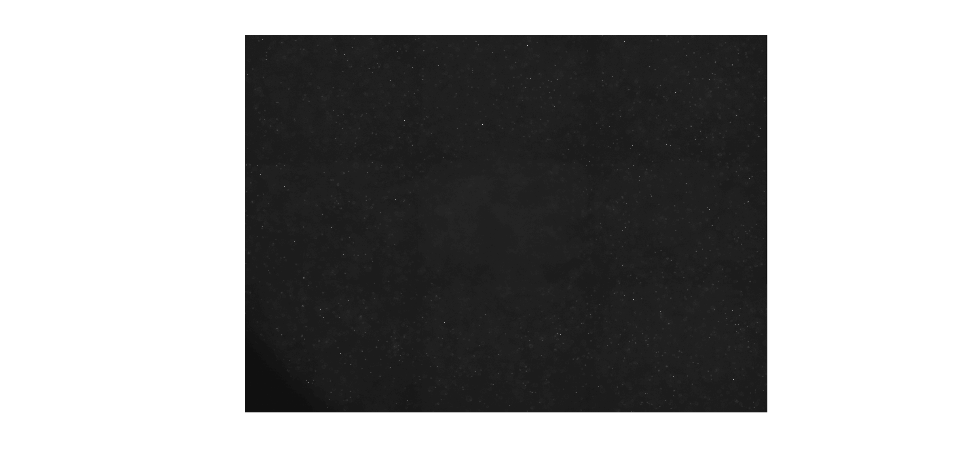


**Supplemental Figure 2:** Image stitching is performed using maximum intensity projection images. Individual 10X image fields are initially aligned based on recorded positioning data. Images are acquired such that there is overlap between the image fields (left), which is used to rigidly register individual fields based on intensity correlation to generate the final stitched image (right).

Following stitching, bead fields at the individual time points are longitudinally co-registered. Supplemental Figure 3 shows the registration steps for the bead field images. 3D point clouds of the bead fields were constructed using the stitched images to represent the centroid position of the fluorescent beads which was extracted using object detection from binarized images. Rigid registration was performed using the 3D point clouds and each imaging time point was longitudinally co-registered to the 24-hour time point. The uncommon z-planes between the z-stacks at each imaging time point, due to microplate repositioning between imaging acquisitions, were eliminated using the point cloud registration. Supplemental Figure 4 shows the results of registration and image processing for the MCTS images. The bead field rigid registration transformations were applied to the MCTS image for each time point and uncommon z-planes across all time points were removed. The maximum intensity projection images of the MCTS at each time point were then used for subsequent image processing. To approximate MCTS cellular density, the fluorescent intensity images were normalized from 0 to 1. Using image thresholding techniques, ROIs were created of the MCTS images, including the invasion region into the ECM. Cell density maps were then interpolated onto the triangular finite element mesh and used as inputs to the model.


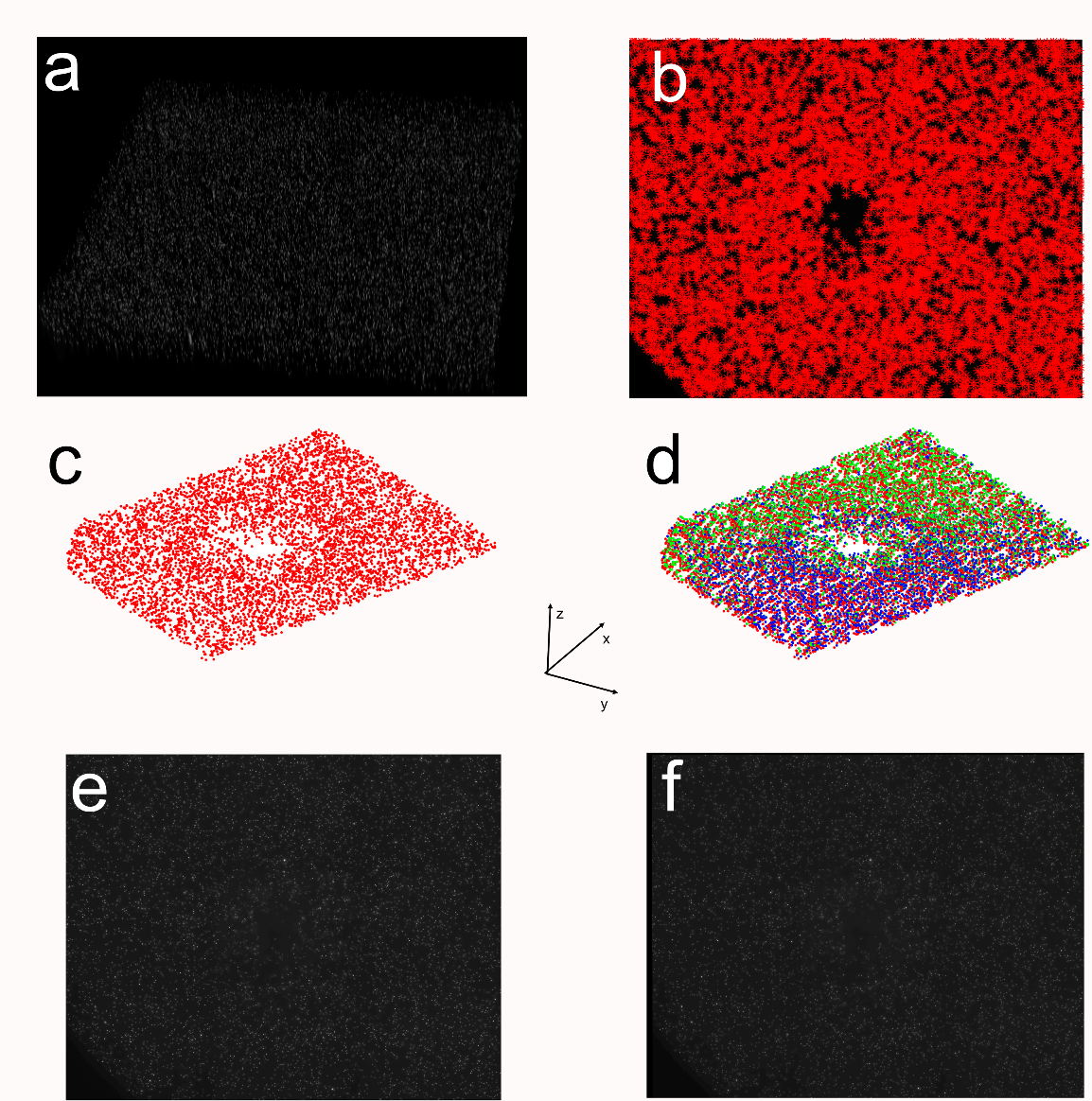


**Supplemental Figure 3:** (a) 3D bead field images are used to longitudinally co-register image acquisition time points. (b) Centroids of each bead location in the XY plane are found from maximum intensity projection images of the bead field. Bead centroid z position is found by identifying the thru-plane maximum intensity pixel for each bead. (c) Point clouds are created for each time point using the centroid position data. (d) Point clouds at each time point are then registered, with alignment shown before and after registration with the target point cloud displayed in red, the source point cloud displayed in blue, and target point cloud registered to the source point cloud displayed in green. The rigid registration transformation matrix is then applied to the original (e) max projection image to create the (f) registered max projection image.


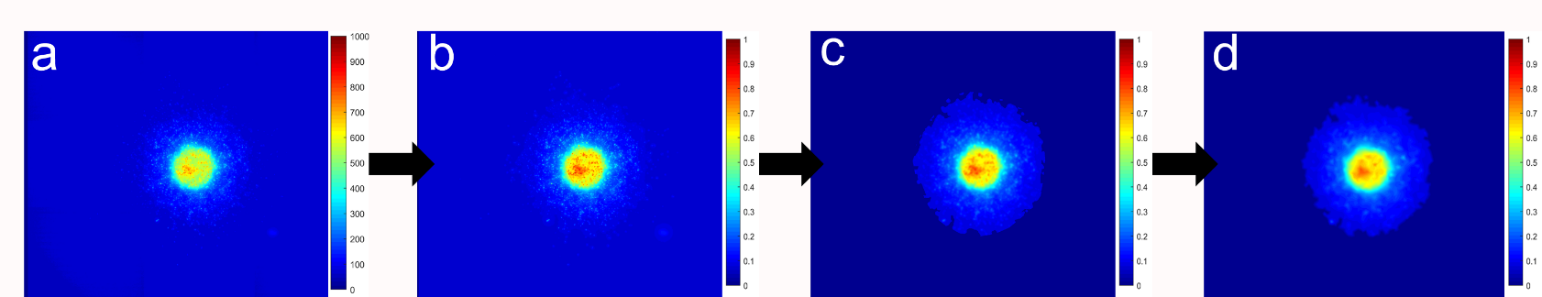


**Supplemental Figure 4:** MCTS image processing follows the following steps for images at each time point: (a) maximum intensity projection images are created from the common z planes for all time points, (b) spheroid fluorescent intensity is normalized to a scale from 0 to 1 to estimate cellular density, (c) an ROI of the spheroid is created using image thresholding techniques, and (d) the cell density map is interpolated onto the finite element mesh.
